# Epidemiology of toxoplasmosis: role of the tick *Haemaphysalis longicornis*

**DOI:** 10.1186/s40249-016-0106-0

**Published:** 2016-02-20

**Authors:** Yongzhi Zhou, Houshuang Zhang, Jie Cao, Haiyan Gong, Jinlin Zhou

**Affiliations:** Key Laboratory of Animal Parasitology of Ministry of Agriculture, Shanghai Veterinary Research Institute, Chinese Academy of Agricultural Sciences, Shanghai, 200241 China; Jiangsu Co-innovation Center for Prevention and Control of Important Animal Infectious Diseases and Zoonoses, Yangzhou, 225009 China

**Keywords:** Tick, *Haemaphysalis longicornis*, *Toxoplasma gondii*, Transmission, Epidemiology

## Abstract

**Background:**

*Toxoplasma gondii* infection is mainly caused by ingestion of water or food that is contaminated with oocysts excreted by cats, or by eating raw meat containing *T. gondii* tissue cysts. However, oral transmission does not explain the common occurrence of toxoplasmosis in a variety of hosts, such as herbivorous animals, birds, and wild rodents. Little information exists on the maintenance of *T. gondii* parasites in nature and routes of transmission to domestic and wild animal hosts. Therefore, this study evaluated the role of *Haemaphysalis longicornis* ticks in the epidemiology of toxoplasmosis.

**Methods:**

The real-time polymerase chain reaction (qPCR) technique was used to detect the presence of *T. gondii* DNA in ticks collected from the field. To observe the amount of dynamic changes of *T. gondii* in the tick’s body and its infectivity, microinjection of green fluorescence parasites was performed. Under laboratory conditions, we evaluated if *H. longicornis* ticks were infected with *T. gondii* and their potential to transmit the infection to other hosts using traditional parasitological methods coupled with molecular detection techniques.

**Results:**

The infection rates of *T. gondii* parasites among field-collected adult and nymph *H. longicornis* ticks were 11.26 % and 5.95 %, respectively. *T. gondii* can survive and remain infective in a tick’s body for at least 15 days. We found that blood feeding of infected ticks did not transmit *T. gondii* to hosts, however, ingestion of infected ticks may be a transmission route between ticks and other common hosts.

**Conclusion:**

The *T. gondii* infection in ticks could serve as a reservoir for toxoplasmosis transmission.

**Electronic supplementary material:**

The online version of this article (doi:10.1186/s40249-016-0106-0) contains supplementary material, which is available to authorized users.

## Multilingual abstracts

Please see Additional file 1 for translations of the abstract into the six official working languages of the United Nations.

## Background

*Toxoplasma gondii* is an obligate intracellular protozoan parasite with a broad host range that includes most bird species and virtually all mammals [1], including humans [2]. It is primarily of medical importance for pregnant women and immunocompromised patients such as those with HIV/AIDS [3, 4]. *T. gondii* may also show veterinary importance as it is causes toxoplasmosis in domestic animals, e.g. neonatal infections and abortion in sheep and goats [3].

Members of the cat family are definitive hosts of *T. gondii*; sexual reproduction of parasites occurs in the small intestine of cats, resulting in the production of oocysts. Infection is mainly caused by ingestion of water or food that is contaminated with oocysts excreted by cats, or by eating raw meat containing *T. gondii* tissue cysts. Although oral transmission has been regarded as the main route of infection [4, 5], it does not explain the common occurrence of toxoplasmosis in a variety of hosts, such as herbivorous animals, birds, and wild rodents. These creatures are improbable to have become infected orally by eating meat or cat feces [6]. Currently, limited information is available on the survival of *T. gondii* in natural settings and its transmission to other warm-blooded hosts. These unresolved issues in toxoplasmosis epidemiology need to be addressed.

Some authors have suggested additional transmission routes of *T. gondii*, including transmission by blood-sucking arthropods [7–10]. Ticks, which are blood-sucking vector arthropods, can carry many kinds of microorganisms while feeding on blood from hosts. However, they may not be able to transmit all of the microorganisms they ingest. DNA of the protozoan parasite *T. gondii* was recently found in field-collected unfed ticks [7, 11], and there have also been reports of the presence of *T. gondii* in naturally infected hard ticks [12, 13]. Suspected cases of human toxoplasmosis were described following tick bites in 1965 [10], and several previous reports have claimed the possibility of experimental transmission with *T. gondii* by ticks [8, 9]. However, some scientists are doubtful that *T. gondii* infection is able to propagate in bodies of poikilothermic animals [3, 14]. The dispute is centered on the fact that the possible role of ticks in the transmission of toxoplasmosis might mainly come from limitations in methodology. However with the progress achieved in research techniques, it is possible to determine the role of ticks in the epidemiology of toxoplasmosis.

The tick *Haemaphysalis longicornis* is distributed mainly in East Asia and Australia [15]. As the dominant tick in China, *H. longicornis* is distributed widely both in the north and south of the country. In this study, we firstly investigated natural *T. gondii* infection in ticks using the real-time polymerase chain reaction (qPCR) technique. We then took the transgenic RH/green fluorescence protein (GFP) strain of *T. gondii* as the marked parasite to evaluate if our sample of *H. longicornis* ticks was infected with *T. gondii*, as well as the ticks’ potential to transmit the infection to other hosts.

## Methods

### Detection of *T. gondii* DNA in field-collected *H. longicornis* ticks

The parthenogenesis tick *H. longicornis* was collected at three sites in the suburbs of Qingdao, Shandong province, China. Ticks were collected between April and October 2012 on three occasions, by flagging. They were then separated into larvae, nymph, and adult ticks, and placed into separate test tubes, where they were stored live until the DNA was extracted. Adults and nymphs were examined singly; larvae were pooled into groups of 20. The total DNA from the tick samples were extracted using a QIAAmp® DNA Mini Kit (Qiagen, MA, USA), according to the manufacturer’s instructions. Quality and concentration of the DNA samples were determined using a spectrophotometer (NanoDrop Technologies, DE, USA). The purified DNA was diluted in distilled water for subsequent PCR reactions. To detect *T. gondii* infection in the ticks, the qPCR technique for the 529-bp target of *T. gondii* was carried out, as previously described elsewhere [16]. To confirm the authenticity of the target sequence, partial positive samples of qPCR were selected to amplify the whole 529-bp target by regular PCR followed by sequencing, as previously described [17].

### Survival and infectivity of *T. gondii* in a tick’s body

The transgenic RH/GFP strain of *T. gondii* (kindly provided by Dr. Xuan, Obihiro University of Agriculture and Veterinary Medicine, Obihiro, Japan) was maintained in human foreskin fibroblasts cells cultured in Dulbecco’s Modified Eagle Medium (Gibco® DMEM), with 10 % of fetal bovine serum(FBS) at 37 °C in a 5 % CO_2_ incubator. Purification of tachyzoites was performed, as described previously [18]. Parasites and cultured cell debris were washed several times in cold phosphate-buffered saline (PBS), and the resulting pellets were resuspended in cold PBS and passed through a 27-gauge needle and a 5.0 μm pore filter (Millipore Corp., Billerica, MA, USA). The *H. longicornis* ticks were maintained in our laboratory. A single engorged female was used to establish the tick colony. A colony of parthenogenesis *H. longicornis* ticks was initiated from one engorged female collected from a deer at the Shanghai Wildlife Park, China. Ticks were reared in a dark incubator at 25 °C, with 92 % relative humidity and fed on a New Zealand white rabbit. After three generations under laboratory conditions, the tick colony was established at the Shanghai Veterinary Research Institute, Chinese Academy of Agricultural Sciences, Shanghai, China [15].

Parasite injection was performed as previously described with some modifications [19]. The injections were done using 10 μL microcapillaries (Drummond Scientific, Broomall, PA, USA) drawn to fine-point needles using a micropipette puller (Narishige, Tokyo, Japan). The needles were then loaded onto a microinjector (Narishige, Tokyo, Japan). *T. gondii* (10^3^) in 0.5 μl of PBS and 0.5 μl of the buffer alone were microinjected from fourth coxae into the haemocoel of unfed adult ticks fixed on a glass slide with adhesive tape in experimental and control groups, respectively. Injected ticks were incubated for three, five, seven, 10, 15, 20, and 30 days at 25 °C, and then DNA was purified from pools (10 ticks) of injected ticks for qPCR detection. The 529-bp DNA fragment in *T. gondii* can be used as a quantitative target for parasite numbers [16, 17]; the relative amount of the target of the 529-bp element of *T. gondii* compared to the tick actin gene (tick house-keeping gene) is regarded as the marker of the number of parasites in ticks. After microinjection, the tick’s body lyses were also observed for live parasites using a fluorescence microscope.

Infectivity of *T. gondii* in ticks was confirmed as follows: Ticks, previously injected with *T. gondii,* were homogenized (groups of 10 adults) in 1.5 ml of normal saline (0.9 % NaCl) containing penicillin and streptomycin. Ticks, previously injected with buffer PBS, were homogenized as controls. Mice (BALB/c, eight weeks old) were injected (0.5 ml/mouse, three mice) intraperitoneally with the tick homogenate in antibiotic solution. Injected mice were observed daily. When clinical symptoms occurred in mice that were killed, such as apathy, depression, and abdominal swelling, appeared, peritoneal dropsy was performed to observe for live parasites using a fluorescence microscope.

### Experimentally infecting *H. longicornis* ticks with *T. gondii*

To confirm tick infection of *T. gondii* by normal tick feeding, we studied tick infection after ticks fed on the blood of infected mice hosts. BALB/c mice (eight weeks old) were used in all experiments, and inoculations were performed as described previously [20]. For inoculation, tachyzoites were cultured and purified from host cells and washed twice in PBS. Parasites were then diluted in PBS with 4000 tachyzoites of the RH-GFP strain (in 500 μl). Mice inoculated with PBS alone were the negative controls. One day after inoculation with *T. gondii*, about 50 nymphs were allowed to feed to repletion on one infected mouse (there was a total of five mice). Engorged nymphs were allowed to molt in the dark at 25 °C, 92 % RH. Newly molted adult ticks were collected and used for *T. gondii* qPCR detection.

### Transmission of *T. gondii* to mice and rabbits by infected ticks

Adult *H. longicornis* ticks*,* infected with *T. gondii* by microinjection or by blood feeding, were used in the transmission experiments. Mice and rabbits served as animal hosts. Twenty eight-week-old BALB/c mice were used in the two tick groups (infection via microinjection or by blood-feeding tick). Four adult *H. longicornis* ticks were allowed to feed to repletion on each mouse. Four New Zealand white rabbits (2.5 kg weight) were used for each of the other two tick groups. Forty adult *H. longicornis* ticks were allowed to feed to repletion on each rabbit. One, 10, and 20 days following tick drop-off, blood was collected for qPCR detection, and sera from the individual mice and rabbits were analyzed using recombinant SAG2 with 1:5000 anti-immunoglobulin G (IgG) antibody [21]. One-month post tick drop-off, blood, heart, lung, spleen, liver, brain, and peritoneal fluid of mice and rabbits were taken and divided between microscopic observation for green *T. gondii* and 529-bp based quantitative PCR for *T. gondii* [15]. Mice were used for blind passages, in which the subsequent batches of mice were inoculated with homogenized organs from the sacrificed mice or rabbits [22].

### Transmission of *T. gondii* to mice by ingestion of infected ticks

*H. longicornis* nymphs, engorged on *T. gondii* infected mice, were collected and allowed to molt to the adult stage. Adult ticks infected with *T. gondii* were homogenized in pools (10 adults), each in 0.5 ml of normal saline (0.9 % NaCI) containing penicillin and streptomycin. Twenty BALB/c mice (eight weeks old) were force-fed the ticks containing the antibiotic solution homogenate. Mice were observed daily and when clinical symptoms appeared in the mice that were killed, such as apathy, depression, and abdominal swelling, peritoneal dropsy was then performed using a fluorescence microscope. Real-time PCR was performed as the above mentioned method [15].

### Animal care and ethics

The animals used for the experiments were treated following the approved guidelines of the Animal Care and Use Committee of the Shanghai Veterinary Research Institute.

## Results

### Detection of *T. gondii* infection in field-collected *H. longicornis* ticks

The infection rates of *T. gondii* parasites among field-collected adult, nymph, and larval *H. longicornis* ticks were 11.26 % (34/302), 5.95 % (5/84), and 0 % (0/36), respectively, using qPCR detection. This demonstrates that adult ticks are most likely to be infected with *T. gondii*; meanwhile, no infected larvae were found. Selected positive samples were amplified by regular PCR for the 529-bp target. Products were sequenced and were identical to the sequences of the *T. gondii* 529-bp gene that is stored in GenBank.

### Survival and infectivity of the *T. gondii* infection in ticks following microinjection

**Fig. 1:**
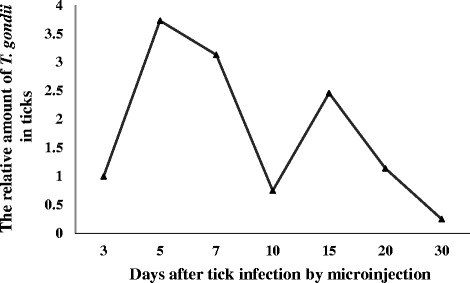
Variation of the numbers of *T. gondii* in ticks at different times after infection by microinjection. The relative amount of *T. gondii* in ticks was showed by copies of the 529-bp fragment of *T. gondii* DNA against copies of the tick actin gene. The 2^-△Ct^ method was used to calculate relative abundance

**Fig. 2:**
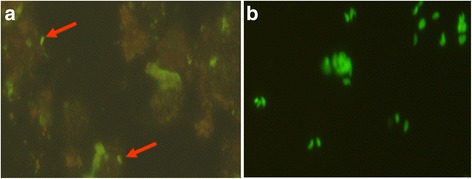
Survival of *T. gondii* in tick’s body and its infectivity to mice. **a**: arrows show the green fluorescence RH-GFP parasites observed in the lyses of a *H. longicornis* tick’s body on day seven, after microinjection; **b**: green fluorescence RH-GFP parasites in peritoneal dropsy of dead mice inoculated with homogenate of injected ticks

The relative amount of the *T. gondii* 529-bp target against the tick actin gene is presented in Fig. 1. The results indicate that *T. gondii* can survive in a tick’s body for extended time periods, but the relative amount of *T. gondii* in ticks seems to show a downward trend from day five to 30. Over the course of 30 days, the *T. gondii* infection had two peaks (on day five and 15) in *H. longicornis* ticks. The green fluorescence RH-GFP parasites were observed in the lyses of a tick’s body on day three, five, seven, 10, and 15, after microinjection. Figure 2a shows the green fluorescence RH-GFP parasites in the lyses of *H. longicornis* tick’s body on day seven, after microinjection.

The infective ability of *T. gondii* in ticks on different days following the injection was tested by inoculating mice intraperitoneally with tick homogenate. *T. gondii* in *H. longicornis* ticks on day three, five, seven, 10, and 15 after injection infected and killed the mice, but the mice in the control group stayed alive. The green fluorescence RH-GFP parasites were also observed in the peritoneal dropsy of mice inoculated with homogenate of lyses of *H. longicornis* tick’s body on day seven after microinjection (see Fig. 2b).

### Experimental infection of ticks with *T. gondii*

Naturally, ticks take blood from the host animal and then molt into the next stage. To confirm tick infection of *T. gondii* by normal tick blood feeding, we evaluated existent *T. gondii* infection in adult *H. longicornis* ticks, which developed from nymphs feeding on infected mice. The amount of engorged nymphs in each mouse varied from 28 to 34. Results showed a high infection rate (mean = 38.71 %) of adult ticks developed from engorged nymphs; the infection rates of tick groups feeding on different mice ranged from 28.57 % to 48.48 % (see Table 1). These results demonstrate that *T. gondii* can be transmitted to ticks by ticks feeding on the blood of infected mammal hosts.

**Table 1:** Tick infection by blood feeding in moue infected with *T. gondii*

Mice fro tick feeding	Number of ticks	Number of positive ticks	Positive rate of ticks
A	28	11	39.28 %
B	33	16	48.48 %
C	35	10	28.57 %
D	25	8	32 %
E	34	15	44.12 %
Total	155	60	38.71 %

### Transmission of the *T. gondii* infection to mice and rabbits by infected tick feeding

Four rabbits for transmission test by ticks infected with microinjection or blood feeding respectively, there is no successful transmission; in the same way, 20 mice for transmission test by ticks infected with microinjection or blood feeding respectively, there is also no successful transmission. Neither in mice nor in rabbit host experiments, were the specific antibodies or nucleotide or green fluorescent parasites detected. Blind passages of mice were taken and did not found positive result.

### Transmission of *T. gondii* to mice by ingestion of infected ticks

In this experiment, 13 of 20 mice were infected based on observation of green fluoresced parasites and qPCR detection in peritoneal dropsy. The seven non-infected mice did not become positive for *T. gondii* in the two-month post-treatment period. These results indicate that ingesting ticks infected with *T. gondii* can lead to mammals acquiring a natural infection.

## Discussion

*H. longicornis* ticks are mainly distributed in East Asia and Australia, and transmit a wide range of pathogens including bovine theileriosis (*Theileria* spp.), bovine babesiosis (*Babesia ovata*), canine babesiosis (*Babesia gibsoni)*, human rickettsiosis (*Rickettsia japonica*), and an emerging tick-borne zoonosis (severe fever with thrombocytopenia syndrome) [23–26]. Our study showed that *T. gondii* DNA were detected in *H. longicornis* ticks at rates of 11.26 % and 5.95 % in adult and nymph ticks, respectively. This result is similar to the infection rate of *T. gondii* in the *Ixodes ricinus* tick, which is mainly distributed in Europe [11]. These unfed ticks from the field are derived from molted engorged larva and nymph ticks; therefore they are no contamination from host animal blood. This indicates the natural infection or survival of *T. gondii* in the *H. longicornis* tick. This study found that adult ticks are most likely to be infected with *T. gondii*; meanwhile, no infected larvae were found. The different levels of presence of *T. gondii* at different developmental stages of ticks might be associated with the frequency with which blood-feeding ticks feed.

The survival of the *T. gondii* infection in *H. longicornis* ticks was studied by microinjection of a *T. gondii* strain exhibiting green fluorescence. *T. gondii* can survive and maintain infectivity in *H. longicornis* ticks for at least 15 days, although other tick species may differ. Peaks of the *T. gondii* infection in *H. longicornis* ticks at different times suggest parasite growth, which contrasts with the present theory that *T. gondii* infection propagates only in warm-blooded hosts [3, 27]. It is necessary to explore this with further experiments.

We validated that ticks infected with *T. gondii* could originate from feeding on the blood of infected mammal hosts under laboratory conditions. This may explain previous observations of ticks naturally infected with *T. gondii* [7, 11–13].

It had been previously claimed that *T. gondii* could be experimentally transmitted to animals by ticks [8, 9]. However, we did not observe the successful transmission of *T. gondii* by blood-feeding adult ticks. The results of this study suggest that toxoplasmosis might not belong to a tick-borne disease, although transmission by infected larvae and/or nymph ticks cannot be ruled out.

However, ingestion of infected ticks can be an effective method for transmission of certain pathogens. For instance, the apicomplexan protozoan parasite *Hepatozoon canis* is transmitted by ingestion [28]. The study demonstrated that ticks become infected when they ingest *T. gondii*, which is present in mammalian blood, and retain *T. gondii* following molting. When mammalian hosts ingest an infected tick, the *T. gondii* infection can be transmitted. This transmission route resembles traditional oral transmission (consumption of raw meat containing infective *T. gondii*) [3, 27]. Oral transmission of ticks may explain the common occurrence of *T. gondii* in a variety of hosts, such as herbivorous animals, wild rodents, and birds [6]. It is important to determine the prevalence of *T. gondii* infection in ticks to establish whether they could represent a significant infection source for toxoplasmosis.

## Conclusion

Our results indicate that *H. longicornis* ticks can harbor viable *T. gondii* parasites, and ingestion of these infected ticks can represent a secondary mechanism of *T. gondii* transmission between ticks and common hosts.

## Additional file

Additional file 1:
**Multilingual abstracts in the six official working languages of the United Nations.** (PDF 381 kb)

## Abbreviations

FBS: fetal bovine serum
GFP: green fluorescence protein
IgG: immunoglobulin G
PBS: phosphate-buffered saline
qPCR: real-time polymerase chain reaction
