# Supplementary material for: Epidemiology of toxoplasmosis: role of the tick Haemaphysalis longicornis
Source: Infect Dis Poverty. 2016 Feb 20;5:14. doi: 10.1186/s40249-016-0106-0 (PMC4761159; doi:10.1186/s40249-016-0106-0)
Supplement: Additional file 1: — Multilingual abstracts in the six official working languages of the United Nations. (PDF 381 kb) [file 40249_2016_106_MOESM1_ESM.pdf]

Translation of the abstract into the six official working languages of the United Nations

الانتشار الوبائي لمرض التوكسوبلازما (داء المقوسات): دور القراد *Haemaphysalis longicornis*

يونج تشى تشو، هوه شوانج تشانج، جي جاو، هيان جونج، جيلين تشو

ملخص

خلفية: تنجم عدوى *Toxoplasma gondii* أساسا بسبب تناول ماء أو طعام ملوث ببيض التوكسوبلازما الذي تواجد في مخلفات القطط، أو عن طريق تناول لحوم نيئة تحتوي على *T. gondii* في الأكياس النسجية. ومع ذلك، لا يفسر الانتقال عن طريق الفم طرق الانتقال الشائعة لداء المقوسات في مجموعة متنوعة من العوائل، مثل الحيوانات العاشبة والطيور والقوارض البرية. توجد معلومات قليلة عن تواجد طفيليات *T. gondii* في الطبيعة وطرق انتقالها لعوائلها من الحيوانات الداجنة والبرية. ولذلك، أجريت هذه الدراسة لتقييم دور قراد *Haemaphysalis longicornis* في الانتشار الوبائي داء المقوسات.

الأساليب: تم استخدام تقنية تفاعل البلمرة المتسلسل اللحظي (qPCR) للكشف عن وجود *T. gondii* DNA في القراد الذي تم جمعه من أماكن تواجده. لمراقبة مقدار التغيرات الديناميكية لـ*T. gondii* في جسم القراد والعدوى بها، تم حقن الطفيليات حقنا مجهريا بومضان أخضر. تحت ظروف المختبر، قمنا بتقييم إذا أصيب قراد *H. longicornis* بـ*T. gondii* وقدرته على نقل العدوى إلى عوائل أخرى باستخدام أساليب دراسة الطفيليات التقليدية إلى جانب تقنيات الكشف الجزيئي.

النتائج: معدلات إصابة بطفيليات T. gondii بين القراد البالغ والحوريات من نوع *H. longicornis* الذي تم جمعه من أماكن تواجده كانت 11.26٪ و 5.95٪ على التوالي. يمكن لـ*T. gondii* البقاء على قيد الحياة والبقاء قادرا على العدوى في جسم القراد لمدة 15 يوما على الأقل. وجدنا أن التغذية بالدم للقراد المصاب لم تنقل T. *gondii* للعوائل، ومع ذلك، ابتلاع القراد المصاب قد يكون طريق انتقال بين القراد والعوائل المشتركة الأخرى.

الخلاصة: عدوى *T. gondii* في القراد يمكن أن تكون بمثابة مكمن لانتقال داء المقوسات.

Translated from English version into Arabic by Mahmoud Sami, through

TRANSLATORS
WITHOUT BORDERS

长角血蜱在弓形虫流行病学中的作用

周勇志，张厚双， 曹杰，龚海燕， 周金林

摘要

引言：宿主感染弓形虫主要通过摄入被猫粪中卵囊污染的饮水或食物，或者吃了含有弓形虫包囊的生肉。不过，经口感染并不能合理解释许多动物宿主的弓形虫病高感染率，如食草动物，鸟类和野生啮齿动物。弓形虫在自然界生存和如何传播到这类家畜和野生动物的研究很少。因此，本试验对长角血蜱在弓形虫病流行病学中的作用进行研究。

方法：应用定量 PCR 方法对野外采集的长角血蜱进行弓形虫核酸的检测。为了观察弓形虫在蜱体内的数量动态变化和感染性，用显微注射的方法将含有绿色荧光蛋白标记的弓形虫注射到蜱体内。实验室条件下，利用传统寄生虫学技术和分子检测手段，我们评估了弓形虫感染长角血蜱的可能性，以及从长角血蜱传播弓形虫到其他宿主的可能途径。

结果：野外采集的长角血蜱成蜱和若蜱的弓形虫感染率分别为11.26%和5.95%。弓形虫在长角血蜱体内至少可以存活15天并保持感染性。我们发现，感染了弓形虫的长角血蜱并不能通过吸血途径传播弓形虫，但动物宿主可通过吞食含有弓形虫的长角血蜱而感染。因此，吞食感染弓形虫的蜱可能是动物宿主感染弓形虫

的重要途径。
结论：蜱是弓形虫传播的储存宿主。

Translated from English version into Chinese by Zhou Jinlin,

Épidémiologie de la toxoplasmose : rôle de la tique *Haemaphysalis longicornis*

Yongzhi Zhou, Houshuang Zhang, Jie Cao, Haiyan Gong, Jinlin Zhou

Résumé

Contexte : l’infection par *Toxoplasma gondii* est principalement causée par l’ingestion d’eau ou d’aliments contaminés par des ovocytes excrétés par des chats ou par la consommation de viande crue contenant des kystes tissulaires de *T. gondii*. La transmission orale n’explique néanmoins pas la présence courante de la toxoplasmose dans une variété d’hôtes, tels que des animaux herbivores, des oiseaux et des rongeurs sauvages. Peu d’informations sont disponibles sur le maintien de parasites *T. gondii* dans la nature et les voies de transmission aux animaux hôtes domestiques et sauvages. C’est pourquoi cette étude a été menée afin d’évaluer le rôle des tiques *Haemaphysalis longicornis* dans l’épidémiologie de la toxoplasmose.

Méthodes : la technologie de réaction en chaîne par polymérase quantitative en temps réel (qPCR) a été utilisée pour détecter la présence d’ADN de *T. gondii* dans des tiques collectées sur le terrain. Une micro-injection de parasites de couleur verte fluorescente a été réalisée afin d’observer la quantité de changements dynamiques du *T. gondii* dans le corps de la tique et son infectiosité. Dans des conditions de laboratoire, nous avons évalué si les tiques *H. longicornis* étaient infectées par le *T. gondii* et nous avons procédé à l’examen de leur potentiel de transmission de l’infection à d’autres hôtes par l’intermédiaire de méthodes traditionnelles de parasitologie associé à des techniques de détection moléculaire.

Résultats : les taux d’infection de parasites *T. gondii* parmi des tiques adultes et de nymphes *H. longicornis* collectées sur le terrain atteignaient respectivement 11,26 % et 5,95 %. Le *T. gondii* peut survivre et conserver son infectiosité dans le corps d’une tique pendant au moins 15 jours. Nous avons découvert que l’alimentation en sang de tiques infectées n’a pas entraîné la transmission du *T. gondii* aux hôtes. Cependant, l’ingestion de tiques infectées peut constituer une voie de transmission entre les tiques et d’autres hôtes courants.

Conclusion : l’infection par *T. gondii* chez les tiques pourrait servir de réservoir de transmission de la toxoplasmose.

Translated from English version into French by Eric Ragu, through

TRANSLATORS
WITHOUT BORDERS

Эпидемиология токсоплазмоза. Роль клеща *Haemaphysalis longicornis*

Yongzhi Zhou, Houshuang Zhang, Jie Cao, Haiyan Gong, Jinlin Zhou

Аннотация

Базовая проблематика. Заражение *Toxoplasma gondii* по большей части обуславливается приемом воды или пищи с ооцистами, выделенными с кошачьими испражнениями, или употреблением сырого мяса, содержащего тканевые цисты *T. gondii*. Однако оральный путь передачи не объясняет общие случаи токсоплазмоза у целого ряда хозяев, например, у травоядных животных, птиц и грызунов. Мало информации о существовании паразитов *T. gondii* в природе и путях заражения домашних и диких животных-хозяев. Таким образом, это исследование дает оценку роли клещей *Haemaphysalis longicornis* в эпидемиологии токсоплазмоза.

Методы. Для выявления ДНК *T. gondii* у клещей, собранных в поле, применялся метод полимеразной цепной реакции в реальном времени (кПЦР). Для наблюдения за количеством динамических изменений *T. gondii* в теле клеща и его инфекционности проводилась процедура микроинъекции паразитов, маркированных зеленым флуоресцентным белком. В лабораторных условиях мы оценили, были ли инфицированы клещи *H. longicornis* паразитами *T.gondii* и определили их потенциал для передачи инфекции другим хозяевам, используя традиционные паразитологические методы в сочетании с методами молекулярной детекции.

Результаты. Зараженность паразитами *T. gondii* среди собранных в поле взрослых клещей и нимф *H. longicornis* — 11,26% и 5,95% соответственно. Паразиты *T. gondii* могут выживать и обладать инфекционными свойствами в организме клеща в течение минимум 15 дней. Мы обнаружили, что зараженные клещи во время укуса не передают *T. gondii* хозяевам, однако, проглатывание инфицированных клещей может быть путем передачи возбудителя от клещей к хозяевам.

Вывод. Клещи, инфицированные *T. gondii*, могут служить источником для передачи токсоплазмоза.

Translated from English version into Russian by Anna Romanenko, through

TRANSLATORS
WITHOUT BORDERS

Epidemiología de la toxoplasmosis: el papel de la garrapata *Haemaphysalis longicornis*

Yongzhi Zhou, Houshuang Zhang, Jie Cao, Haiyan Gong, Jinlin Zhou

Resumen

Información de referencia: La infección de *Toxoplasma gondii* se debe principalmente a la ingestión de agua o comida contaminada con oocistos excretados por gatos, o por comer carne cruda que contenga cistos de tejido de *T. gondii*. No obstante, la transmisión oral no explica la incidencia común de la toxoplasmosis en diversos huéspedes, como animales herbívoros, aves y roedores salvajes. Existe poca información sobre el mantenimiento de parásitos de *T. gondii* en la naturaleza, así como sobre las vías de transmisión a huéspedes, tanto animales domésticos como salvajes. De ahí que este estudio haya evaluado el papel de las garrapatas *Haemaphysalis longicornis* en la epidemiología de la toxoplasmosis.

Métodos: Se utilizó la técnica de la reacción en cadena de la polimerasa en tiempo real (qPCR) para detectar la presencia de ADN de *T. gondii* en garrapatas recogidas sobre el terreno. Para observar la cantidad de cambios dinámicos de *T. gondii* en el cuerpo de la garrapata y su infectividad se procedió a microinyectar parásitos por

fluorescencia verde. Bajo las condiciones del laboratorio, evaluamos si las garrapatas de *H. longicornis* se infectaban con *T. gondii* y su potencial para transmitir la infección a otros huéspedes, utilizando para ello métodos parasitológicos tradicionales junto con técnicas de detección molecular.

Resultados: Las tasas de infección de parásitos de *T. gondii* entre adultos recogidos sobre el terreno y ninfas de garrapata *H. longicornis* fueron de 11,26% y 5,95%, respectivamente. *T. gondii* puede sobrevivir sin infectar en el cuerpo de una garrapata durante un mínimo de 15 días. Averiguamos que las garrapatas infectadas que se alimentaron de sangre no transmitieron *T. gondii* a los huéspedes; sin embargo, la ingestión de garrapatas infectadas podría ser una vía de transmisión entre las garrapatas y otros huéspedes comunes.

Conclusión: La infección de *T. gondii* en garrapatas podría servir como reserva para la transmisión de la toxoplasmosis.

Translated from English version into Spanish by SergioLorenzi, through

TRANSLATORS WITHOUT BORDERS
